# [Retracted] NCTD elicits proapoptotic and antiglycolytic effects on colorectal cancer cells via modulation of Fam46c expression and inhibition of ERK1/2 signaling

**DOI:** 10.3892/mmr.2023.13078

**Published:** 2023-08-29

**Authors:** Shiqiang Zhang, Yun Yang, Yunwei Hua, Chen Hu, Yi Zhong

Mol Med Rep 22: 774–782, 2020; DOI: 10.3892/mmr.2020.11151

Following the publication of this paper, it was drawn to the Editor's attention by a concerned reader that the GAPDH control western blotting data shown in Fig. 2C were strikingly similar to data appearing in different form in other articles written by different authors at different research institutes. Moreover, certain of the western blotting data shown internally within Fig. 4E and F appeared to be strikingly similar, even though the experiments portrayed in these Figure parts were intended to show the results obtained from different cell lines.

Owing to the fact that the contentious data in the above article were already under consideration for publication, or had already been published, prior to its submission to *Molecular Medicine Reports*, and based on an overall lack in the confidence in the data, the Editor has decided that this paper should be retracted from the Journal. The authors were asked for an explanation to account for these concerns, but the Editorial Office did not receive a reply. The Editor apologizes to the readership for any inconvenience caused.

